# A novel research method for workshops and co-production of interventions: using a secret Facebook group

**DOI:** 10.1186/s40814-020-00711-0

**Published:** 2020-11-02

**Authors:** Audrey Buelo, Alison Kirk, Ruth Jepson

**Affiliations:** 1 Scottish Collaboration for Public Health Research and Policy, School of Health in Social Science, University of Edinburgh, Edinburgh, UK; 2 Physical Activity for Health Group, School of Psychological Sciences and Health, University of Strathclyde, Glasgow, UK

**Keywords:** Facebook, Co-production, Intervention, Physical activity, Gestational diabetes

## Abstract

**Background:**

Co-production of interventions is reliant on good communication and consensus between participants and researchers, but attending in-person meetings and workshops is hard for time-constrained groups such as new mums, who may be geographically dispersed without reliable transport. Discussions with a lay advisory group resulted in the decision to hold a workshop over a secret Facebook group. The aim of this study was to test the feasibility of a secret Facebook group for co-production activities. In the example presented, the population was women with previous gestational diabetes, the topic was physical inactivity, and the purpose was to develop an acceptable physical activity intervention.

**Methods:**

The researchers created a secret Facebook group with content similar to an in-person workshop that sequentially progressed to develop a programme theory for an intervention. The researcher posted 1–2 times per day for 14 days, and members of the group were invited to comment and discuss the content. Feasibility and acceptability of the group were analysed using Facebook analytics and a post-workshop survey.

**Results:**

Twenty-one participants took part. In total, 521 comments were provided in response to 18 posts of varying types (average = 28.9 comments per post). The total word count of participant comments was 21,142 words. The workshop was viewed positively, with 20 of 21 participants saying they liked the workshop “somewhat” or “a great deal”, and felt the group was a safe and open environment to share opinions. When asked if they would take part in something like this again, 15 of 21 said “Yes”. Participants mentioned the format was convenient; it allowed them to reflect on their experiences, and they liked helping research progress. Those who say “maybe” said it was difficult finding time and depended on what else was going on.

**Conclusion:**

Using a secret Facebook group as a method of co-production or as a workshop in the research process is a feasible and acceptable method. Social media holds significant potential for co-production and involvement in research for populations who are geographically dispersed or time-constrained, with an uncommon condition or other circumstances where *in-person* meetings are either not appropriate or not possible.

**Supplementary Information:**

**Supplementary information** accompanies this paper at 10.1186/s40814-020-00711-0.

## Introduction

At its core, co-production is defined as the contribution of service users to service provision—though the amount of contribution varies within and between fields [1]. In regard to intervention and service development, it refers to moving beyond the typically top-down approach of viewing end-users as passive recipients of a service to viewing them as active participants with assets to help in the development and delivery of the service or intervention [2]. Co-production of complex public health interventions is seen as an increasing necessity in intervention development for improving acceptability and sustainability of interventions [3]. It involves key stakeholders working alongside researchers to co-develop an intervention that is feasible, acceptable, and sustainable by taking account of contextual factors (e.g. need, culture, geography, preferences, resources). Co-production in intervention development typically involves in-person workshops or consultations, and face-to-face interviews or focus groups in which intervention materials are developed and tested with those who will receive, deliver, or resource the intervention, and feedback provided on various aspects. Whilst the use of co-production has increased steadily since the 1970s [1], the challenges of co-production are not insignificant. It can be time-consuming and difficult to balance competing priorities of involved stakeholders and is not suitable in every situation [2, 4]. In-person co-production can be an efficient, convenient method of working together, which aids relationship building, but is not suitable for all demographics. In particular, creating a workshop or focus group at a time and location that suits everyone is challenging when working with populations that have an uncommon condition, are geographically dispersed, or have limited opportunities to gather.

### The internet as a tool for co-production

The internet has dramatically changed modern modes of communication and connected those who may otherwise never have an opportunity to interact. Asynchronous remote communities (ARC) are an expanding method of research that allows participants to interact in focus group-style research online and at a time that suits them [5]. Researchers post questions, activities, or prompts, and participants are encouraged to respond and interact with each other at their convenience [6].

Facebook is an online social networking site developed in 2004. Originally intended for university students, Facebook has now exploded in popularity around the world—with nearly 1.59 billion daily users [7]. It allows users to get in touch with people with similar interests, backgrounds, and experiences instantaneously. The capabilities of Facebook now extend far past keeping in touch with friends and family. People use it as a main source of news, forming connections around similar hobbies, interests, and illnesses, and for self-promotion [8, 9].

Groups are a feature of Facebook that allows like-minded individuals together to connect and share experiences. Facebook groups have varying levels of privacy: An *open group* is viewable to anyone on Facebook and not restricted to members of the group. *A closed group* has content only viewable by the group members, who are able to join through an approval process. Anyone can see the existence of a closed Facebook group. However, a *secret group* is not viewable by anyone except those in the group—who are able to join only by direct invitation [10]. It is private and hidden in searches; those who are not members of a secret Facebook group cannot see the existence or the content of the secret Facebook group [11]. This type of privacy holds particular benefit for research, as confidentiality and anonymity are ethically important in internet-mediated research.

Previous studies have conducted focus group-style research in private or secret Facebook groups [5], with the purpose of conducting a needs analysis or collecting data in a more focus group-style to understand a problem. Research groups at the University of Indiana and the University of Edinburgh have particular experience with asynchronous remote communities, or focus groups occurring online in which participants are not online at the same time, and have published widely on the topic—primarily short reports with recommendations for future research and lessons learned [12, 13]. However, none of the studies we have found to date uses asynchronous remote communities, particularly in a secret Facebook group, to co-design an intervention for future testing.

### Our research: secret Facebook groups as a tool to co-produce interventions with hard-to-reach groups

This paper describes a method of co-production and intervention development specifically designed to overcome barriers of in-person research methods: online, secret Facebook groups. In this paper, we will describe the development and use of the secret Facebook group in a dispersed group of participants with a relatively rare health condition and discuss the benefits and limitations for research. This secret Facebook group study was part of a larger intervention development research study, using the Six Steps in Quality Intervention Development (6SQuID) framework [3]. The 6SQuID framework is based on six steps: (1) identify a public health problem and its causes, (2) clarify the modifiable and non-modifiable causal factors, (3) identifying the theory of change (what causal pathways to interrupt and how), (4) identify the theory of action (how to deliver the intervention), (5) test and refine on a small scale, and (6) collect sufficient evidence of effectiveness to justify more rigorous evaluation and implementation [3].

The 6SQuID framework was used to structure a research project to develop a physical activity intervention for a group of women at high risk of type 2 diabetes. The secret Facebook group was used to inform steps 2 through 4 of 6SQuID. In step 1 of 6SQuID, the researcher conducted a mixed-method systematic review [14] and aimed to conduct focus groups with the target audience to understand the problem but faced difficulties in recruitment and attendance—as previous research has noted with this group as well [15]. Twenty-five interviews were subsequently carried out with success to understand the problem of physical inactivity. However, steps 3 and 4 of 6SQuID require group consensus to determine causal pathways to interrupt and what intervention components are acceptable to the target population. A new method of data collection was needed that allowed for group consensus, remote access, and flexibility.

### Partners in co-production

The population group for our study was women with a history of gestational diabetes, a disease that affects 4.4% of pregnancies worldwide and significantly increases risk of type 2 diabetes later in life [16, 17]. Physical activity was the target behaviour of this intervention development study as it is a beneficial tool for type 2 diabetes prevention for women with previous gestational diabetes as well. A US-based prospective cohort study of 4554 women with a history of gestational diabetes found that for every 100 min increase of moderate-vigorous physical activity performed per week, there was a 9% reduced relative risk of T2DM onset [95% CI, 0.88–0.94], even after adjusting for diet and BMI [18].

A lay advisory group composed of three women with previous gestational diabetes^1^ was consulted about an ideal method of co-production; they favoured a secret Facebook group for several reasons including convenience, high existing use, and ease of use of the website and phone application. Previous research has shown that 81% of mothers use Facebook, and 56% of these mothers check the platform several times a day [19]. Additionally, online focus groups and in-person focus groups have been shown to be comparable in terms of quality of content collected [20].

## Aims and objectives

The aim of the study was to test feasibility and acceptability of using a secret Facebook group for co-production. This paper describes the approaches used and their benefits and limitations.

### Feasibility objectives

1. Is the secret Facebook group a feasible and acceptable method for co-producing a physical activity programme for women with previous gestational diabetes, from the perspective of both the participant and the researcher?
2. How frequently will this method be used by participants?
3. What content will generate the most engagement?

## Methods

This paper reports on a feasibility study using mixed methods including post-study survey and descriptive analysis of responses. As mentioned, it is a study nested within a larger study developing an intervention.

### Recruitment

The in-person interviews previously conducted provided an initial source of participants, as the participants in this group consented to being contacted for future research. Thus, the majority of participants (16/21) had previously had face-to-face or telephone interviews with the researchers from June to September 2018. New participants (5/21) were recruited from a Facebook group that is specifically for women with current or previous gestational diabetes. The researchers received permission from the Facebook group administrator to post a flyer advertising the study. Interested members reached out to a study-specific email address to receive further information and access to an online information sheet and consent form.

All participants were living in the UK during the study but were geographically dispersed.

### Sample size

The sample size was determined based on previous studies of asynchronous remote community research, with studies ranging from 13 participants to 48 participants [21–25]. As the secret Facebook group takes place over a longer period of time, a larger group of participants can be accommodated in comparison to an in-person focus group or an online synchronous focus group [25].

### Content development

The Facebook group content was developed to address steps 2 through 4 of the 6SQuID framework: to clarify modifiable and causal factors of the problem, develop a theory of change, and begin to develop a theory of action for a physical activity intervention [3]. A theory of change is the process by which change comes about for individuals, groups, and communities—it asks: how are we going to change this behaviour? To create a theory of change, we first performed a situation analysis (commencing in the previous in-person interview portion) to understand contributing factors to physical inactivity for this group and developed a fishbone diagram with participants to explain key barriers and facilitators to physical activity in their daily lives (see Supplementary file 1 and 2).

The current study built on the information gained during the interviews to understand modifiable and important factors to understand which factors can be changed with the greatest scope for improvement in their physical activity. The researcher presented the fishbone diagram of barriers to physical activity to the secret Facebook group participants to understand which factors they felt were the most important and significant barriers in their lives. This allowed the researchers to be able to understand which factors needed to be modified to change their physical activity. The researcher then analysed this information to begin to develop a theory of how to change their behaviour: potential intervention ideas, settings, timing, and other components were presented back to participants for views of acceptability and feasibility.

### Running the workshop

The workshop took place over a 15-day period in May to June 2019, the length of which was based on previous research [26]. To action the 6SQuID steps, the lead researcher created posts for the Facebook page that sequentially and cumulatively led to the steps above being completed. Both the lay advisory group and co-authors reviewed the drafts of the posts. The lead researcher aimed to use several different formats of Facebook posts including text, photo series, polls, and embedded videos for variety to keep participants engaged. Emojis (digital icons to display an idea or emotion) were used by the researcher when posting in the group in a similar manner to participants; the researcher mimicked the style of writing by participants to encourage an informal atmosphere. Two lay advisory group members were also involved as participants and co-facilitators to encourage discussion if posts did not have any responses. The researcher posted one to two times per day, with 2 days in the middle of the workshop that were “catch-up” days (day 8 and day 10). Messages and content were typically posted between 15:30 and 17:30 GMT, as suggested by the lay advisor group, as mothers would be returning from work and may have a short break before their evening meal. The researcher checked the group several times each day during the workshop to ask follow-up questions and clarifications and moderate if necessary. Participants were able to comment on the researcher posts with their opinions and also were able to generate their own posts. Participant posts had to be approved by the researcher to ensure they were appropriate to the group.

### Ethical considerations

The British Psychological Society published ethical guidelines for internet-mediated research in 2017 encompassing 4 main principles: (1) respect autonomy, privacy, and dignity; (2) maintain scientific integrity; (3) social responsibility; and (4) increase the benefits whilst reducing the harms [27]. Confidentiality, anonymity, and identity verification were specific ethical issues considered in developing the online workshop. The nature of social media existing online holds inherent confidentiality risks. Facebook ultimately possesses the information on the website, and participants were informed of this in the consent process. Their data is not innately private because of the platform, but in terms of privacy of what one posts on social media being viewed by others, a secret Facebook group is clearly a private space—participants should feel able to write freely without concern that what they are saying is viewable to those who are not in the group. In contrast to confidentiality involving how the researcher manages and uses private information, anonymity involves obscuring identifiable information for participants and can be used to maintain confidentiality of identifying information [28, 29]. The part of qualitative analysis of social media that involves a significant risk to anonymity is in disseminating the results [27]. For research using open and online internet forums, the risks to anonymity are higher, as participant quotations can be traced back to the original source and can potential identify the participant. However, this is less of a risk in a secret Facebook group: a secret group is not searchable on Facebook or any search engine, and details given in the group are only viewable by group members. As such, anonymity is preserved barring any data breaches through participants sharing or Facebook security lapses. When participants were first added to the secret Facebook group (after providing informed consent), they were required to read through the “Group Rules”, in which one of them provided explicit privacy instructions: “Respect Everyone’s Privacy and Confidentiality. Being part of this group requires mutual trust. Everyone is welcome to share sensitive information, thoughts and feelings in this group, but what’s shared in the group should stay in the group”, to ensure that participants understood the privacy and confidentiality implications of the research.

Identity verification holds two primary considerations in social media research: (1) Are participants who they say they are? (2) Does the person that a participant portrays in a Facebook group accurately represent their real self? As “Facebook profile page amounts to a blank canvas on which each user has free reign to construct a public or semi-public image of him- or herself”, it is possible the participants that the researcher has not met before may not fit the eligibility criteria (p. 213) [30]. However, this research is not concerned with who the participant actually is, but rather with each participant’s opinions and perceptions on intervention ideas. It is possible that people may say they approve or disprove of something that they do not actually approve or disprove of (in real life)—thus harming the data collected—but this is a risk in any research with people [10].

Informed consent was taken online in a Qualtrics survey, as studies have shown that an online consent form provides equal comprehension compared to a written consent form [31]. Participants were emailed a link to a survey that laid out the same information as a written participant information sheet and consent form, but clicked “agree” after each consent statement to indicate consent, and then provided identifying details to verify their identity including year of birth, year diagnosed with gestational diabetes, name, and Facebook profile name and link. Participants were informed during the consent process prior to the workshop commencing how to adjust their privacy settings of their Facebook profile—this allowed them to restrict what other participants in the secret Facebook group could see of their profile. The online study was approved by the University of Edinburgh Health in Social Science ethics committee.

### Evaluation of the online workshop

An evaluation plan was developed to explore the feasibility and acceptability of this method to generate a theory of change and theory of action for the intervention. The key components of a feasibility study include acceptability, practicality, demand, implementation, adaptation, integration, expansion, and limited efficacy, based on Bowen and colleagues’ research around important aspects of feasibility studies [32]. Acceptability and practicality are most relevant for this study, and how they were assessed is described in detail in Table 1. Demand can be assessed using the Facebook analytics because it shows actual use. Implementation will be based on analytics and the researcher’s own experiences of executing the plan. Practicality, integration, and limited efficacy were assessed based on the overall results of the workshop, whereas adaptation and expansion should be explored in future research.

**Table 1:** Evaluation plan of the secret Facebook group workshop. Based on Bowen and colleagues’ “How we define feasibility studies” paper [32]

Aspects and design	Aim of evaluation	Details
**Acceptability** Survey—quantitative qualitative	Are recipients and deliverers satisfied with the method? Does it feel appropriate?	Survey questions involve assessing participant enjoyment, if they felt it was a safe environment to share thoughts, the timing and quantity of posts, among other questions.
**Practicality** Survey—quantitative qualitative	To what extent can the workshop be carried out with intended participants using existing resources? Are they able to carry out intervention activities?	
**Implementation** Memo-ing and overall success of workshop	1. Can it be successfully delivered to participants in some defined but not fully controlled context? 2. What kind of resources is needed? 3. What factors affect implementation? 4. What is the speed and quality of implementation?	Researcher’s own experiences (daily memo-ing and reflecting on process)
**Demand** Facebook analytics	1. How likely is this method to be used? 2. What content generated the most and least interaction?	Actual use of the workshop by participants. SocioGraph to measure: most commented posts, most reacted posts, average words per comment, and ratio of “seen by” per “number of comments”

### Data analysis

To analyse the data, participants were asked to suggest their own pseudonyms at the end of the survey for analysis and report-writing; these pseudonyms are reported here. Descriptive analyses were performed for the survey questions, as there were no comparison groups. Simple counts were performed to determine the number of comments in total and for each post. For the purposes of developing an intervention, the researcher conducted thematic analysis based on Clarke and Braun’s thematic analysis methodology (not reported in depth here) [33].

## Results

Twenty-one participants were recruited into the study from 16 May to 28 May 2019. Two women were recruited from the lay advisory group, 16 were recruited from the previous interview stage of the research, and 5 were recruited from a closed, gestational diabetes-specific Facebook page. The average age of participants was 35.8 years (age range 25–47), and the average time since last diagnosis of gestational diabetes was 2.4 years (range < 1 to 10 years). Participants were based throughout Scotland. At the end of the workshop, one participant informed us that she was 6 weeks pregnant (an exclusion criterion of the study), but her comments and responses were still included in the Facebook page as she had not found out she was pregnant until that point.

### Responses

Table 2 provides a summary of the posts and responses. In total, 521 comments were provided in response to 18 posts of polls, video, text, or photos for an average of 28.9 comments per post. The total word count of all comments (excluding the researcher’s responses and comments) was 21,142 words. Thirty-two comments (6.1% of all participant comments) were participants directly interacting with each other, without the researcher facilitating the conversation.

**Table 2:** Summary of Facebook posts and interaction with participants

Date of post	Aim of post	Format of post	Number of likes/comments (excluding researcher)
Day 1	To explain the purpose of the workshop, who is involved, and what participants can expect over the coming 2 weeks	Text	19 likes/2 comments
Day 1	For participants to introduce each other and share some meaningful details about their lives to feel more comfortable	Text with photo	3 likes/29 comments
Day 2	To clarify what is meant by a “physical activity programme”	Text	1 comment
Day 2	To decide how we want to define physical activity moving forward	Poll with images	21 votes/14 comments
Day 3	To review the factors that women said made PA easier in the interviews; to gain consensus on modifiable and important factors	Text with 8 photos (see Fig. 1 for example image)	2 likes/139 comments
Day 4	What are participant’s views on self-care? How important do participants feel is it to look after themselves?	Text with photo	3 likes/33 comments
Day 5	Follow-up question about self-care	Text with photo	3 likes/18 comments
Day 5	To review the factors that women say make them less likely to do PA; to gain consensus on the biggest barriers to PA	Text with 8 photos	1 like/158 comments
Day 6	To decide how we want to define “success” in terms of the physical activity programme that is being developed	Poll (text)	1 like/39 votes/9 comments
Day 7	To access acceptability of her workout method and thoughts on a PA intervention	Text with linked video	2 likes/23 comments
Day 8	No content, day off for catching up	Text with photo	5 likes/2 comments
Day 9	To clarify what the focus of the programme should be (PA vs mental health/PA vs PA/diet etc.)	Poll (text)	2 likes/18 votes/12 comments
Day 9	Summary of barriers and facilitators post: to ensure that what I have pulled out of the discussion is what they agree with (validation)	Text with two photos summarising top 10 barriers/facilitators (see Fig. 2 for example image)	14 likes/4 comments
Day 10	No post, catch-up day	N/A	N/A
Day 11	To understand how to address the barrier of childcare access	Text with photo	1 like/27 comments
Day 12	Intervention development, getting feedback on mental health intervention (mindful self-compassion)	Text with imbedded video	6 likes/27 comments
Day 13	Intervention development, getting feedback on the intervention setting	Poll (text)	2 likes/23 votes/11 comments
Day 14	To assess interest in taking part in subsequent in-person workshop	Poll (text)	3 likes/24 votes/8 comments
Day 15	Asking participants to take part in end-of-workshop survey	Text with linked survey	9 likes/4 comments

*PA* physical activity

**Fig. 1:**
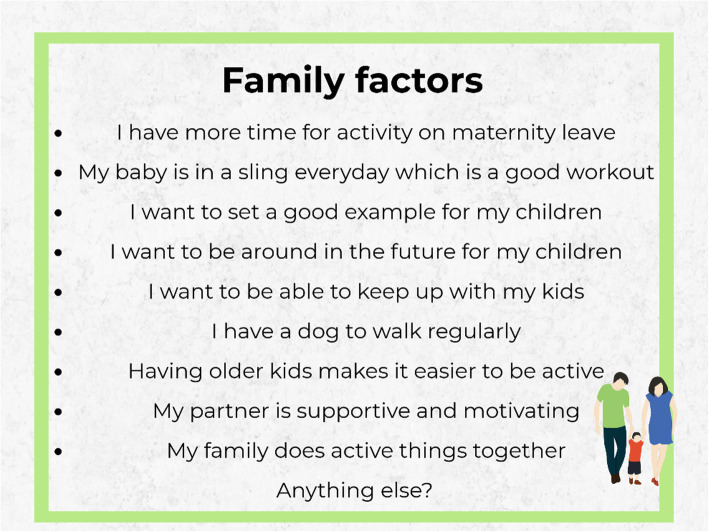
Example image of family-related facilitators to physical activity (identified during previous interview stage). Participants were asked to comment on the image and list 1–3 factors within this theme that were most important to them

**Fig. 2:**
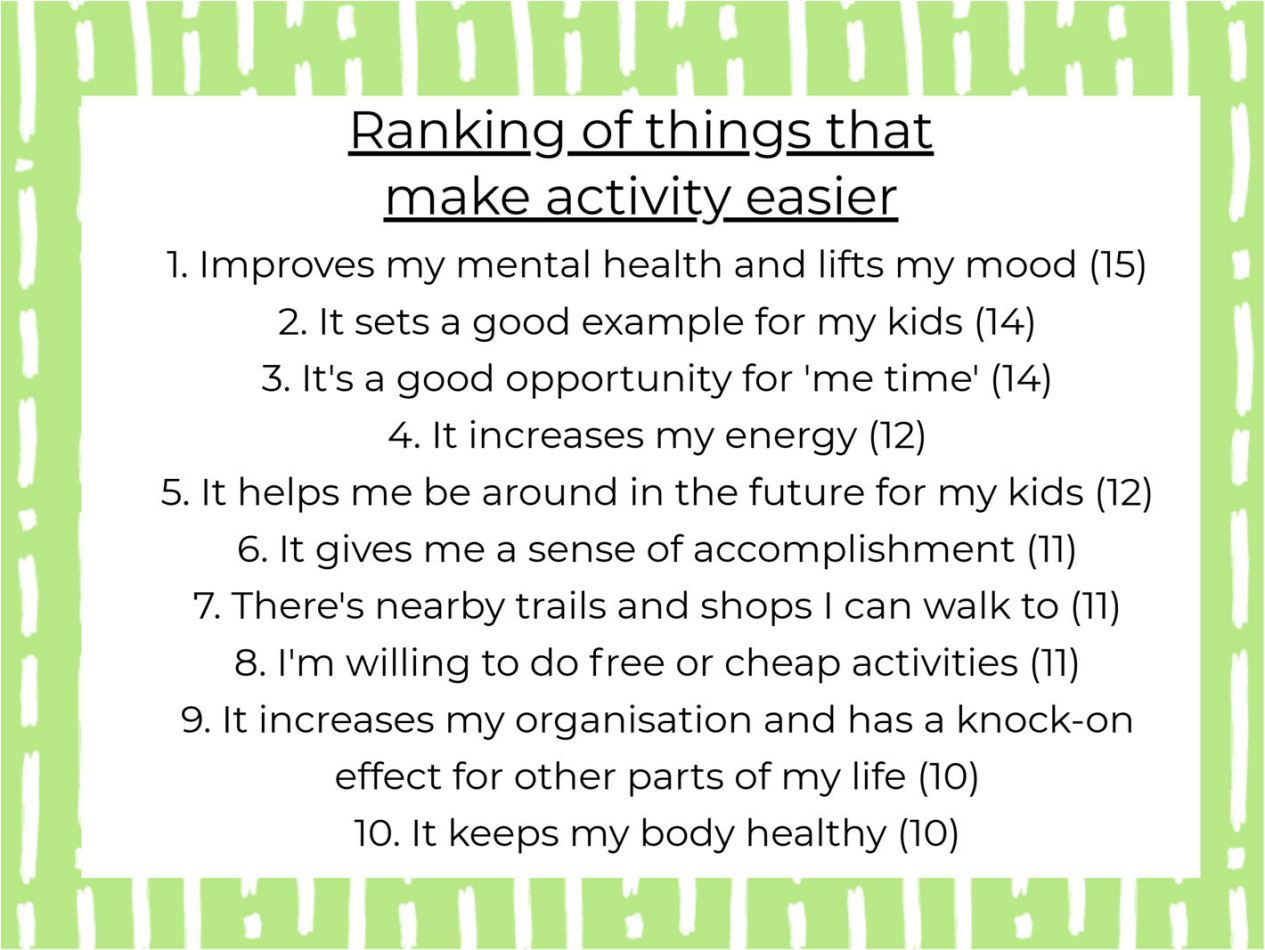
The ranking of the top 10 factors women said made physical activity easier. Women then comment on this post with their approval and any additional comments they wanted to share

The most popular posts (with the highest number of comments) were the two posts containing a series of images listing barriers and facilitators to physical activity—with 138 and 158 comments, respectively. Having conducted previous qualitative interviews on factors influencing physical activity, the researcher had generated a list of barriers and facilitators to physical activity and grouped them according to the social determinants of health model [34]. Participants were asked to choose 1–3 factors within each photo series that they thought had the greatest influence on their physical activity levels. For example, for family-related facilitators to physical activity (Fig. 1), a participant commented, “I want to set a good example for my daughter, and be around in the future for her. I was out and about with her regularly in the sling on maternity leave, not nearly so much now I’m back at work” (Mia). The majority of other participants agreed in that being around to see their children grow up was a significant motivating factor. This suggested that emphasising that particular benefit of physical activity (e.g. it is life-lengthening and allows for more healthy years of life to spend with children and family) might resonate with this population more than other facilitators. From tallying the votes for the most important factors from these posts (Fig. 2), it allowed the researcher to generate a “top 10” list for barriers and facilitators, which was critical in understanding what to address in the theory of change.

### Feasibility of the method

From the researcher’s own memos and reflections on the data collection, several lessons and themes were discerned from the online workshop: polls were an easy and popular way of gaining consensus about intervention options. They were efficient and well-answered, with a minimum of 18 votes per poll (average of 25 votes per poll, maximum of 39 votes). The researcher allowed for participants to select multiple options as well as add their own option to the poll and let others vote on it, which aligned with the co-production goals of the research.

### Increasing response rates

Sending out gentle reminders over Facebook messenger to participants who had not participated in the previous 3 days was a useful way of boosting participation rates. Asking for varied methods of input became a useful tool in gathering opinions when the group slowed down slightly in the final days of the workshop. During day 12, the researcher posted a video asking for feedback and asked participants either to comment with their thoughts or to “react” in a certain way to show their views (e.g. thumbs up meant “like”, angry reaction if “I don’t think it’s for me”, and laughing reaction if “unsure”). Reacting in different ways to posts allowed all content to become polls in part, which allowed more time-constrained participants to still have their voices heard.

It was useful for the researcher to check the page often in the few hours after the post to moderate if necessary. At times, someone would post something negative or controversial (nothing significant that warranted further action), but it was helpful to respond quickly to empathise and/or ask for other opinions to ensure the group stayed on track. The lay advisory group members who were both participants and co-facilitators did not end up acting in the latter role as it was not necessary—participants overall answered each post quickly and did not need further facilitation.

At the end of the workshop, the researcher posted a final message thanking participants for taking part and reminding them to take the survey. However, some participants continued posting in the group after the official end of the study—two shared links relevant to the topic of the workshop, and another asked a question related to her gestational diabetes follow-up care. This suggests that the online workshop may have potential to be self-sustaining, as the researcher did not explicitly mention or encourage posting after the culmination of the study.

The results from the end-of-workshop survey are detailed in Table 3. In short, overall, the workshop was viewed positively. It was enjoyed by participants, with 20 of 21 participants (95%) saying they liked the workshop “somewhat” or “a great deal”. The same percentage said they felt the group was a safe and open environment to share opinions, with the dissenting view mentioning her dislike of Facebook more generally. Perhaps, the most important indicator of acceptability was asking participants if they would take part in something similar again, in which case over 70% (*n* = 15) said “Yes”. Participants who said they would take part again mentioned how the format was very convenient to fit into their day and when they had time; it allowed them to reflect on their own experiences, and they liked helping research progress for gestational diabetes. The six participants who said they *may* take part again said it was still difficult finding time to do it, it depended on what else was ongoing in their lives, and one participant suggested it was not as detailed as she anticipated it to be. No participants said they would *not* take part again. Overall, the workshop was acceptable to participants.

**Table 3:** Summary table of the end-of-workshop survey results

Survey question	Main results (%)	Specific comments
Overall, how much did you like participating in the Facebook group?	57.1% Liked somewhat (*n* = 12) 38.1% Liked a great deal (*n* = 8) 4.8% Neither liked nor disliked (*n* = 1)	N/A
Did you feel the group was a safe and open environment for you to share your opinions?	95.2% Yes (*n* = 20) 4.8% No (*n* = 1)	No (*n* = 1): “Facebook in general. I’ve tried to leave it a few times and then I almost didn’t sign up for [this study] as it meant signing into Facebook.” (Rae)
If you did not participate in all of the posts, why not?	N/A, I participated in all: 57.1% (*n* = 12) Didn’t answer question: 14.3% (*n* = 3) I didn’t have time: 4.8% (*n* = 1) I didn’t notice the post: 0% Other reason: 14.3% (*n* = 3) Combination of above reasons: 9.5% (*n* = 2)	Other reasons: “Busy schedule” (Paige) “Booked a last minute holiday” (Rosie) “I fully intend to go back and respond to those I missed, but sometimes just couldn’t get to them on the day they were posted.” (Jennifer) “Bereavement and hospital with child. And Facebook being pretty crap on your phone browser. FB want you to use their app.” (Rae)
On average, how did you feel about the length of each post?	An appropriate length: 95.2% (*n* = 20) Too long: 4.8% (*n* = 1) Too short: 0%	N/A
On average, how did you feel about the time of day that the researcher posted in the group? (typically between 15:30 and 17:30 on weekdays)	The timing of the posts was fine: 85.7% (*n* = 18) I wish she had posted earlier in the day: 14.3% (*n* = 3) I wish she had posted later in the day: 0%	N/A
On average, how did you feel about the number of posts in the group? (typically 1–2 times per day for 2 weeks, with 2 days without posts 1-week in)	I thought the number of posts was fine: 76.2% (*n* = 16) I wish she had posted more often: 14.3% (*n* = 3) I wish she had posted less often: 9.5% (*n* = 2)	N/A
Would you take part in a study like this again?	Yes: 71.4% (*n* = 15) Maybe: 28.6% (*n* = 6) No: 0%	Yes (selected comments): “If it helps someone else then it’s always worth doing” (Shannon) “It was a nice way to do the study - was able to participate at the time that suited me and my schedule each day.” (Poppy) “Was great to be able to dip in and contribute when had time (normally once the kids were in bed) was good to hea[r] other viewpoints and experiences” (Rebecca) “Made me reflect and was nice to see others in same position reflect. Nice to think it was helpful for research too.” (Elizabeth) “I’m always happy to take part in studies to help research into little known conditions such as gd” (Lorena) “Yes, it’s good to know people want genuine experiences to help others in future and it’s nice to meet like-minded mums” (Irene)
		Maybe (selected comments): “It’s hard to get time when you have two kids under 3 as they want all your attention. Then there’s the dog and husband as well it’s hard even to get time to do a survey.” (Yvonne) “The study wasn’t as detailed as I expected it to be” (Emily) “It will depends on what else I have on during the week. I was away for part of the first week and I had to play catch up for some of the posts” (Allie)

## Discussion

The results from this research suggest that co-producing an intervention over a secret Facebook group is a successful approach to collecting consensus and generating ideas to create a physical activity intervention for women with a history of gestational diabetes (refer to Table 4 for a summary). There was clear reach and engagement with the content, as approximately 28 comments per post were generated by participants. The volume and quality of data generated suggest that holding a workshop in a secret Facebook group was a feasible method of data collection, and the results of the end-of-workshop survey suggest it was acceptable to participants. This aligns with other studies showing information was shared equally (or more) freely in an online environment in comparison to in-person discussions [6, 35, 36].

**Table 4:** A summary table of the lessons learned in feasibility of holding an intervention development workshop in a secret Facebook group

Aspects and design	Aim of evaluation	Summary of results
**Acceptability**	Are recipients and deliverers satisfied with the method? Does it feel appropriate?	Attendees of the workshop found it overall an acceptable method.
**Practicality**	To what extent can the workshop be carried out with intended participants using existing resources? Are they able to carry out intervention activities?	As the workshop method uses Facebook asynchronously, a widely used platform by participants, they were able to complete the research posts at their own pace. Participants reported that they were able to complete the majority of activities, though some reported problems with knowing what activities they had completed and others said it was difficult if their lives were particularly busy.
**Implementation**	1. Can it be successfully delivered to participants in some defined but not fully controlled context? 2. What kind of resources is needed? 3. What factors affect implementation? 4. What is the speed and quality of implementation?	1. The results suggest that this method is feasible to be implemented in a defined but not controlled context. 2. The resources needed are primarily researcher time to develop and facilitate the group, and all participants need access to the internet. 3. There are many factors that influence implementation. Researcher time and availability during the workshop influenced participant engagement. Internet access, time zones, and location of participants also affect implementation and should be considered at the design stage. 4. This method can be very quickly implemented if needed, though the authors suggest that time is taken prior to the start of the Facebook group to draft posts and review the appropriateness of the language for your target audience.
**Demand**	1. How likely is this method to be used? 2. What content generated the most and least interaction?	1. This method has high potential to be used to intervention development with hard-to-reach and time-constrained groups. 2. Photo series and polls generated the most interaction, whereas text posts without any questions or prompts generated the least interaction.

The online workshop proved a very useful method to develop a nuanced understanding of the issues over the time period. It did become clear as the workshop progressed what the key issues were regarding physical inactivity in this group. From developing the theory of change, the theory of action quickly followed, and the researcher was able to quickly gather consensus about intervention components by posting in the secret Facebook group, which will be reported in future work. The intervention content is currently under development and will be tested further in 2021.

A key strength of the research was that location was not an obstacle to participation. Participants in this study were spread throughout Scotland—geographic spread and rurality of participants is typically a significant barrier to co-production. Holding an intervention development workshop online vastly increases the input from groups who may not be able to attend face-to-face, due to time, location, or circumstantial constraints. This opens up the possibility of doing research with hard-to-reach groups and allows for a more representative group of participants to take part—thus improving the quality and external validity of the data. Beyond hard-to-reach groups, in light of the COVID-19 pandemic and restrictions on group gatherings, socially distanced measures of data collection such as this one can ensure that research progresses safely.

A further strength of this method was that, although potentially novel in its use, it remained evidence-based in the asynchronous remote community (ARC) literature. Previous research has suggested optimal methods of recruitment (online networks), content (a friendly and informal atmosphere, posting often, similar prompts to in-person focus groups), sample size (between 13 and 48 participants), facilitator role (to respond quickly to questions, post prompts at similar times daily), and analysis method (content analysis), which this study followed closely [5, 10, 12, 13, 25]. Additionally, given that this method allowed for participants to take as much time as needed to consider and respond to a prompt—in contrast to the immediacy of in-person interviews and focus groups—another strength could be increased thoughtfulness of answers. The researchers could also take time to consider responses and ask follow-up questions in a more considered manner than what may occur during in-person qualitative research.

There were a few limitations of this study, mainly related to the use of Facebook. The Facebook algorithm of what content users see on their home feed could have influenced the participation rates of this study. Some participants mentioned in the end-of-workshop survey that if they did not check the Facebook group directly for a few days and wanted to catch-up, it was difficult to find the posts they had and had not already contributed to without scrolling through all of the comments. Also, if participants engaged less with the Facebook group for a few days, the Facebook algorithm would likely reduce the amount they saw the Facebook group on their timeline—further supressing the visibility of the Facebook group content. There are two possible solutions to this: (1) A participant in the end-of-workshop survey mentioned that to ensure that she knew when she had commented on a post, she “liked” the post. This allowed her to see more easily what she had engaged with. (2) Another option could be an external checklist that participants actively mark to signify their participation in each post.

Another limitation comes with the potential negativities of using social media in general—people are reporting efforts to reduce their social media use, with descriptions of it being “time-wasting” and potentially having a negative impact on their mental health [37]. Whilst most of the target population uses Facebook daily, some are trying to limit use. By having this workshop exclusively over Facebook, we may be unintentionally excluding participants who do not want or have access to this type of social media. There are also inherent privacy concerns to the use of Facebook in general. If participants had open and viewable Facebook profiles and elected not to increase the privacy of their profile prior to the workshop, other participants would be able to see personal details of their lives that face-to-face focus groups would not display.

This study found that the use of secret Facebook groups was feasible and acceptable to participants but was unable to determine if it results in a more effective and sustainable physical activity intervention for the target audience. Future studies should explore if this method is feasible and acceptable to other groups of the population and for other research topic areas. As discussed, this demographic was previously active users of Facebook which likely contributed to the high participation rates seen. However, there may be other groups that use different social media or do not use Facebook. Twitter, Instagram, YouTube, and popular new media platforms such as TikTok have different capabilities and target demographics to Facebook and could be useful tools for co-production or workshops for appropriate groups.

## Conclusion

This methodology paper suggests that using a secret Facebook group for running an intervention development workshop is acceptable to participants, is feasible for the researcher to conduct, and generates high-quality, nuanced data. This method holds significant promise in similar future work with geographically remote communities, those who have difficulty travelling or limited time, or those with relatively uncommon diseases or risk factors.

## Supplementary Information

**Additional file 1: Supplementary file 1.** A fishbone diagram demonstrating barriers to physical activity.

**Additional file 2: Supplementary file 2.** A fishbone diagram demonstrating facilitators to physical activity.

## Data Availability

Data sharing is not applicable to this article as no datasets were generated or analysed during the current study.

## Abbreviation

6SQuID: Six Steps in Quality Intervention Development

## Footnotes

1 The lay advisory group was recruited to assist throughout the research project. They were given a modest yearly payment in exchange for their advice and assistance in the research and to offset any travel costs from meetings.
