# Supplementary figures and images for: A novel research method for workshops and co-production of interventions: using a secret Facebook group
Source: Pilot Feasibility Stud. 2020 Nov 2;6:168. doi: 10.1186/s40814-020-00711-0 (PMC7605463; doi:10.1186/s40814-020-00711-0)

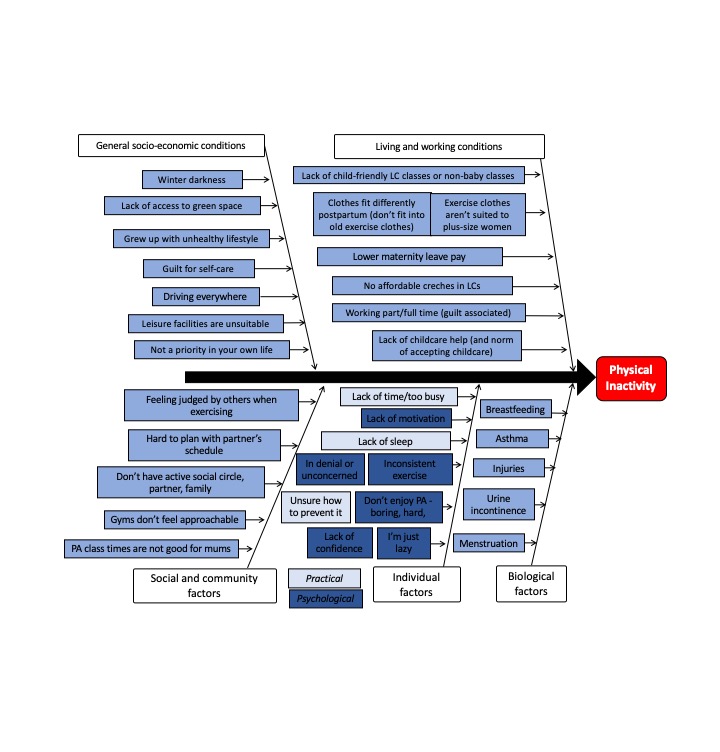

Supplement: Supplementary file 1 — Additional file 1: Supplementary file 1. A fishbone diagram demonstrating barriers to physical activity. [file 40814_2020_711_MOESM1_ESM.jpg]

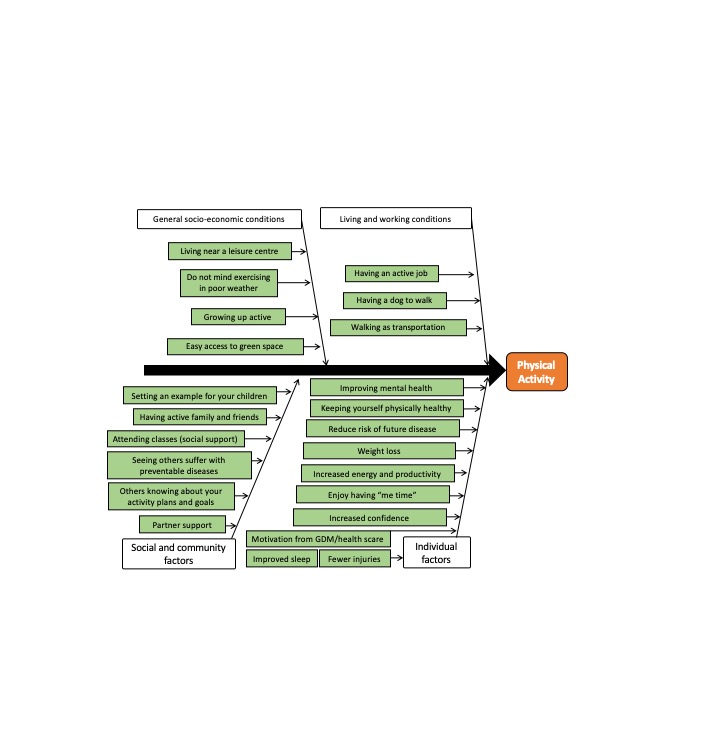

Supplement: Supplementary file 2 — Additional file 2: Supplementary file 2. A fishbone diagram demonstrating facilitators to physical activity. [file 40814_2020_711_MOESM2_ESM.jpg]
